# Patient perspectives of target weight management and ultrafiltration in haemodialysis: a multi-center survey

**DOI:** 10.1186/s12882-021-02399-7

**Published:** 2021-05-20

**Authors:** David Keane, Megan Glyde, Indranil Dasgupta, Claire Gardiner, Elizabeth Lindley, Sandip Mitra, Nicholas Palmer, Louise Dye, Mark Wright, Ed Sutherland

**Affiliations:** 1 Renal Medicine, Leeds Teaching Hospitals NHS Trust, Leeds, UK; 2 Leeds Institute for Cardiovascular and Metabolic Medicine, University of Leeds, Leeds, UK; 3 School of Psychology, University of Leeds, Leeds, UK; 4 University Hospitals Birmingham NHS Foundation Trust, Birmingham, UK; 5 Dietetics, Leeds Beckett University, Leeds, UK; 6 Manchester Academic Health Sciences Centre, The University of Manchester, Manchester, UK; 7 Devices for Dignity, NIHR MedTech & In-vitro Diagnostics Co-operative, Sheffield, UK; 8 Kidney Care UK, Alton, UK

**Keywords:** Haemodialysis, Health surveys, Ultrafiltration, Target weight, Patient participation, Patient knowledge

## Abstract

**Background:**

Decisions around planned ultrafiltration volumes are the only part of the haemodialysis prescription decided upon at every session. Removing too much fluid or too little is associated with both acute symptoms and long-term outcomes. The degree to which patients engage with or influence decision-making is not clear. We explored patient perspectives of prescribing ultrafiltration volumes, their understanding of the process and engagement with it.

**Methods:**

A questionnaire developed for this study was administered to 1077 patients across 10 UK Renal Units. Factor analysis reduced the dataset into factors representing common themes. Relationships between survey results and factors were investigated using regression models. ANCOVA was used to explore differences between Renal Units.

**Results:**

Patients generally felt in control of their fluid management and that they were given the final say on planned ultrafiltration volumes. Around half of the respondents reported they take an active role in their treatment. However, respondents were largely unable to relate signs and symptoms to fluid management practice and a third said they would not report common signs and symptoms to clinicians. A fifth of patients reported not to know how ultrafiltration volumes were calculated. Patients responded positively to questions relating to healthcare staff, though with significant variation between units, highlighting differences in perception of care.

**Conclusions:**

Despite a lack of formal acknowledgement in fluid management protocols, patients have significant involvement in decisions regarding fluid removal during dialysis. Furthermore, substantial gaps remain in patient knowledge and engagement. Formalizing the role of patients in these decisions, including patient education, may improve prescription and achievement of target weights.

**Supplementary Information:**

The online version contains supplementary material available at 10.1186/s12882-021-02399-7.

## Introduction

Decisions around fluid management in haemodialysis (HD) have a significant impact on both short- and long-term outcomes [1–6]. Furthermore, deciding on the planned ultrafiltration volume is the only part of the dialysis prescription that is actively considered at every single session. This centrality and frequency make decisions relating to fluid management incredibly important.

Prescription of fluid removal in haemodialysis involves a number of components. These include dialysis scheduling and interventions like profiling ultrafiltration rates or dialysate sodium concentrations [7, 8], all of which aim to avoid hypovolemia. Arguably the most important aspect is the target weight. Target weight can be defined in a number of ways [9] but aims to prevent hypovolemia and chronic fluid overload, minimise patient symptoms and prevent loss of residual kidney function [5]. Setting the target weight too high will lead to fluid overload which is associated with mortality [10]. Setting the target weight too low leads to rates of fluid removal that exceed what the body can compensate for. As well as leading to end-organ hypoperfusion, the patient will be susceptible to acute symptoms such as intradialytic hypotension (IDH) which is linked to mortality [11, 12].

Target weights are largely managed by clinical assessment, a practice that has remained fundamentally unchanged since the inception of haemodialysis [13]. Technology to assist decision-making has been developed, most promising being bioimpedance spectroscopy, but sufficient evidence to support adoption is lacking [14, 15]. Approaches to managing target weights varies dramatically between and even within renal units, both in the UK and internationally [6, 16].

Achieving target weight is also important. In a study of over 12,000 US patients, 21% regularly had a post-dialysis weight over 2 kg above or below their target weight [17]. Every kg difference between target weight and achieved post-dialysis weight has been associated with a 2.7-fold higher risk of mortality [18].

Despite considerable focus on improving management of fluid removal on HD, the interaction of the patient with decision making has been largely neglected. This is notable. Clinical assessment, the basis of target weight prescription, involves patient reported symptoms while strong clinician-patient partnerships are clearly central to achieving target weights. This study aimed to explore patient perspectives of target weight management and ultrafiltration in a large sample of haemodialysis patients in renal units across the UK.

## Materials and methods

### Study design

We developed a cross-sectional survey which was administered to in-centre HD patients in the UK. The study was approved by the Health Research Authority Research Ethics Committee (reference 17/SC/0596) and reported in accordance with STROBE guidelines [19]. The study was conducted in accordance with the Declaration of Helsinki. Informed consent was indicated by completion of the questionnaire.

### Questionnaire development

The content of the questionnaire was informed by a qualitative study involving 12 haemodialysis patients [20] and relevant literature. Questionnaire items were reviewed with five experts in renal care (including 1 patient).

The questionnaire was piloted with 11 haemodialysis patients using a convenience sample and was then revised accordingly. The final questionnaire consisted of 38 questions (19 Likert, 8 closed questions, 10 questions about demographics and 1 free text box – Additional file 1). Topics covered included patients’ perception of:

- Understanding of target weight
- Impact of poor fluid management
- Involvement in decision making
- Knowledge and reporting practices around intradialytic signs and symptoms
- Impact of bad experiences upon subsequent fluid removal decisions
- Patient perception of how staff understand their fluid management

### Questionnaire reliability & validity

The questionnaire underwent test-retest reliability testing with 20 patients and a 2-week gap between assessments. Nominal data was assessed by Cohen’s kappa, Likert-scale data by weighted Cohen’s kappa. Content validity was assessed by 6 relevant experts (Renal Dietitian, haemodialysis patient in a professional advocacy role, three Nephrologists and Clinical Scientist), using the content validity index [21].

### Participants

The inclusion criteria were in-centre haemodialysis patients, over 18 years old, who could understand and complete the questionnaire which was only available in English. All questionnaires were completed between May 2018 and December 2019. At each renal unit, patients were recruited by convenience sampling. Research nurses were encouraged to offer the questionnaire to all eligible patients dialysing on a particular shift in a particular location to minimise selection bias. However, the ability to approach patients across different shifts and locations was dependent on the practicalities and resource availability at each renal unit. The questionnaires were mostly completed during treatment, with an option to complete at home if preferred. Research nurses facilitated completion of questionnaires when requested but the research nurses were not part of study conception, data input or analysis and were independent from the patient’s clinical care team.

### Data analysis

#### Defining predictor and outcome variables

Categorical predictors with more than two groups were grouped into binary variables (age: 18–65, 66+ years; ethnicity: Caucasian, non-Caucasian; education: up to school age 16, above age 16; years since beginning HD: up to 3, more than 3; daily urine output: less than a cupful, more than a cupful).

Knowledge scores were calculated by summing all correct responses to questions about the causes of intradialytic fluid management signs and symptoms (out of 9). Reporting totals were calculated by summing the number of symptoms patients stated they would report to staff if they were to experience it (out of 7). For the symptom knowledge and reporting totals, missing data, “I don’t know” responses or incorrect responses were inferred as not knowing/not reporting.

#### Factor structure of the questionnaire

Exploratory factor analysis (principal axis factoring with varimax rotation) was used to determine the factor structure of the Likert scale questions. Items were removed if they had poor individual Kaiser-Meyer-Olkin values or did not load substantially on to a factor. The number of factors were extracted according to the scree plot [22] with a Kaiser-Meyer-Olkin statistic of 0.776 (please see Additional file 2 for the list of questions included in each factor, and their factor loadings). Internal consistency reliability was assessed using Cronbach’s α coefficient [23] for all Likert scale questions and for each factor separately.

Weighted factor scores were generated for patients who answered all, or all but 1, question within a factor by multiplying the question factor loading by the raw Likert-scale score for that question, and then calculating the average for all questions within a factor.

#### Predicting patient perspectives and knowledge of fluid management

We used multiple regression to explore relationships between demographic variables and weighted factor, knowledge and reporting scores. Weighted factor scores were also used as predictors for total knowledge and reporting scores but were not used to predict other weighted factor scores [24]. The initial model included all predictors and the most parsimonious model was retained. The models were bootstrapped due to deviations from normality [25].

#### Exploring differences between renal units

Differences in weighted factor, knowledge and reporting scores between renal units were assessed using analysis of covariance (ANCOVA), controlling for demographics and factor scores shown to be significant in the multiple regression models. The models were bootstrapped due to deviations of normality [25].

### Statistical analysis

Little’s test of Missing Completely at Random was non-significant confirmed data was missing completely at random (*p* = 0.07) and pairwise deletion applied [26]. IBM SPSS Statistics for Windows, version 23.0 was used for all analyses.

## Results

### Study cohort

1077 participants completed the questionnaire across 10 UK renal units and the number in each unit is detailed in Table 1. A range of 8–54% of the renal units’ in-centre haemodialysis patients completed the questionnaire, which combined was 26% of all units’ patients. Patient characteristics are detailed in Table 2.

**Fig. 1:**
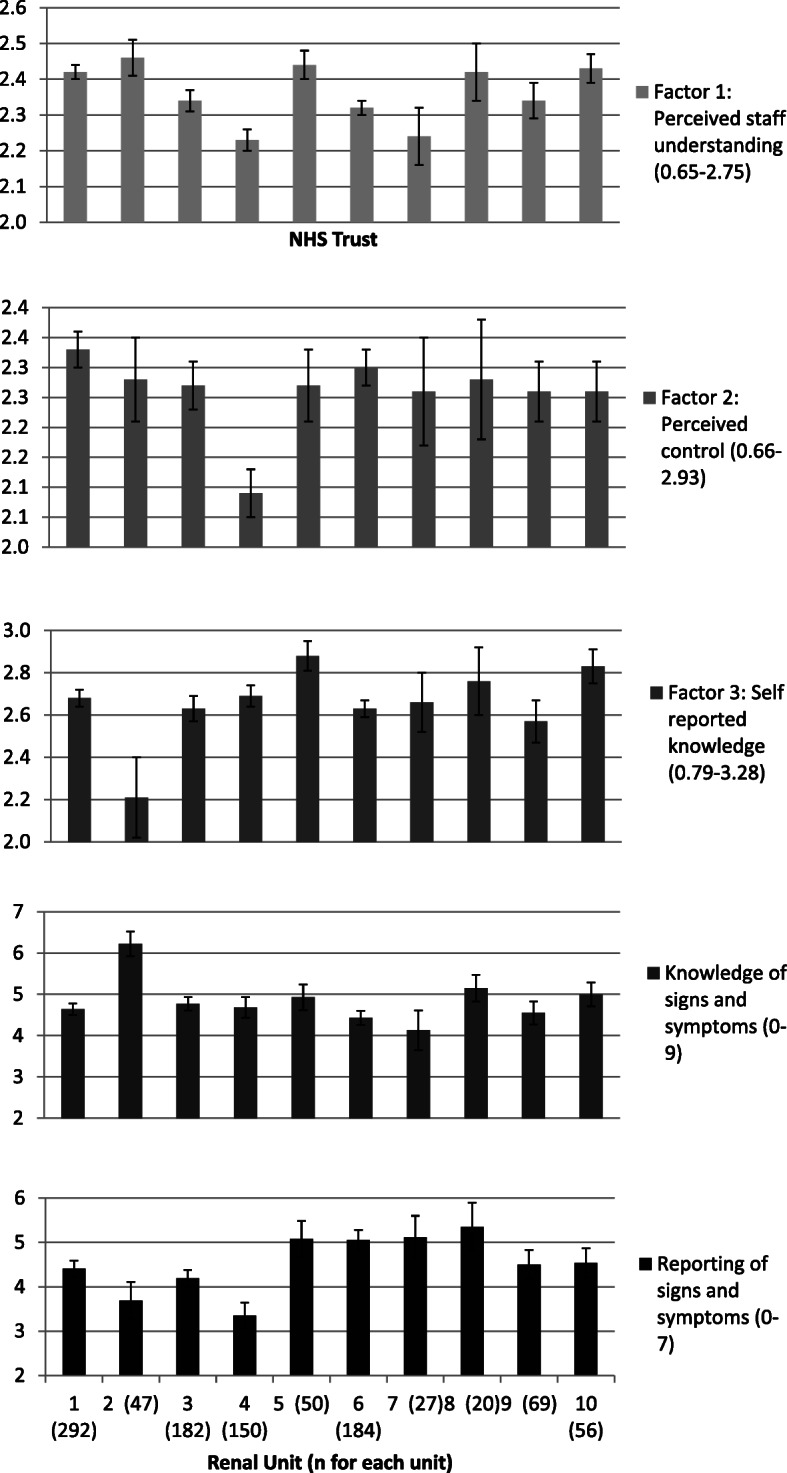
Adjusted means & bootstrapped standard errors for each renal unit after controlling for significant predictors. All differences *p* < 0.05 from ANCOVA models. Number of completed questionnaires at each unit is indicated in the axis label

**Table 1:** Demographic characteristics of patients who responded to the survey

Characteristic	Total n (%)	Range by renal unit (%)
Age (yrs)		
18–25	23 (2%)	0–4%
26–35	40 (4%)	0–6%
36–45	79 (7%)	0–15%
46–55	163 (15%)	9–18%
56–65	235 (22%)	12–25%
66–75	252 (23%)	16–42%
76+	260 (24%)	16–41%
Ethnicity		
White	849 (79%)	72–96%
Black	56 (5%)	0–11%
Asian	119 (11%)	0–18%
Other	16 (2%)	0–3%
Sex		
Male	663 (62%)	52–80%
Female	361 (34%)	10–40%
Education		
Up to school age 16	427 (40%)	29–53%
Above school age 16	410 (38%)	23–50%
Comorbidities		
Heart failure	190 (18%)	8–44%
Diabetes	375 (35%)	20–54%
Dialysis vintage (years)		
< 1	236 (22%)	5–28%
1–3	347 (32%)	26–52%
3–5	177 (14%)	8–30%
5–10	150 (14%)	7–18%
> 10	128 (12%)	6–18%
Urine output		
None	240 (22%)	9–37%
Less than a cupful per day	313 (29%)	24–40%
More than a cupful per day	487 (45%)	37–52%
HD sessions per week		
< 3	30 (3%)	0–8%
3	991 (92%)	78–100%
> 3	26 (2%)	4–19%
Previous kidney transplant		
Yes	184 (17%)	6–22%
No	854 (79%)	71–89%

**Table 2:** Study cohort by renal unit and as a proportion of the total HD population of the unit

Renal unit	Patients included in the study	Total HD patients at the unit^a^	Proportion (%)
1	292	538	54%
2	47	549	9%
3	182	387	47%
4	150	500	30%
5	50	183	27%
6	184	899	20%
7	27	352	8%
8	20	128	16%
9	69	423	16%
10	56	160	35%

^a^Note, not all dialysis locations under the care of each unit were included, leading to the high range in proportions

### Reliability and validity

Following Altman’s classification, test-retest results indicated that 7% of the questions had fair, 41% moderate, 44% good and 7% very good reliability [27]. The questionnaire had content validity with all items having a content validity index score of 0.83 or above [21].

### Patients’ experiences

The majority (79%) of patients felt they in control of their fluid management. Furthermore, patients largely felt that they were asked about (85%) and given the final say (82%) in deciding how much fluid to remove, while 51% felt they took an active role in their treatment or engaged with a shared care program. Sixty six percent (66%) of patients stated they had had an unpleasant experience during dialysis due to having too much fluid removed, and 66% of these patients believed this affected how much fluid they were subsequently willing to have removed.

### Reporting and knowledge of signs and symptoms

The ability to link common signs and symptoms to removing too much or too little fluid varied quite substantially. Only 14% of patients identified that an increased risk of fistula failure could be related to removing too much fluid during dialysis, whereas 59% of patients correctly answered that this could lead to cramping and a rapid drop in blood pressure (Table 3).

**Table 3:** Knowledge and reporting of signs and symptoms; n (%)

Symptom	Correctly identifies if symptom is related to removing too much fluid or having too much fluid in your body	Would routinely report this symptom to staff if this symptom occurred
Swollen tissue	627 (58%)	715 (66%)
Feeling very tired after dialysis	469 (44%)	685 (64%)
Dizziness	623 (58%)	812 (75%)
Increased risk of fistula failure	152 (14%)	–
Shortness of breath	479 (44%)	784 (73%)
Passing less urine	351 (33%)	524 (49%)
Thirst	415 (39%)	466 (52%)^a^
Cramping	634 (59%)	754 (70%)
A rapid drop in blood pressure	634 (59%)	–

^a^Due to printing errors in one of the units, the question relating to whether patients report thirst was incomprehensible and so was removed from analysis, leaving only 893 completed responses for this specific item

Reporting of signs and symptoms also varied, with dizziness most likely to be reported and thirst least likely to be reported.

### Target weight

Twenty percent (20%) of patients reported that they did not know their target weight, yet 76% of patients agreed that they understood how staff use their target weight to work out how much fluid to remove during dialysis. Seventy percent (70%) of patients agreed they would remove more fluid than normal if they were fluid overloaded, however 30% said they would be happy to remain fluid overloaded in order to finish their dialysis session early. Also, 56% of patients considered flexibility in their diet and fluid restrictions as more important than getting to their target weight at each dialysis session.

### Factor structure of the questionnaire

The scree plot suggested 3 factors to the solution: factor 1 “perception of staff understanding of the patient’s fluid management”; factor 2 “patients’ perceived control of their fluid management”; and factor 3 “self-reported knowledge of the long-term effects of poor fluid management”. Higher scores reflect better perceived staff understanding, feeling more in control, or feeling more knowledgeable about the long-term impacts. The questionnaire as a whole as well as each individual factor had good internal consistency (all Cronbach’s α > 0.7).

### Predicting patient perspectives and knowledge of fluid management

The variance explained by the models was small (1.3–18.7%) but in each case significant. Non-Caucasian patients were more likely to rate staff understanding of their own fluid management (factor 1) lower (Table 4). For factor 2, older patients were predicted to score lower on their perception of having control of their fluid management. For factor 3, women, patients who have previously had a kidney transplant, and patients with less urine output were predicted to self-report greater knowledge about the long-term effects of poor fluid management.

**Table 4:** Bootstrapped multiple regression identifying predictors for knowledge and reporting of signs and symptoms. Beta values and 95% bootstrapped confidence intervals are presented in brackets

Predictors & model information	Factor 1: Perceived staff understanding *n* = 837	Factor 2: Perception of control *n* = 941	Factor 3: Self-reported knowledge *n* = 742	Knowledge of signs and symptoms *n* = 697	Reporting of signs and symptoms *n* = 869
Δ R [2]	.01^b^	.02^b^	.03^b^	0.19^b^	.02^b^
Age (66+ years)		−0.07 [− 0.1, − 0.01]^a^		−1.7 [−2.1, − 1.4]^b^	− 0.4 [− 0.7, − 0.1]^a^
Ethnicity (non-Caucasian)	−0.1 [− 0.2, − 0.04]^b^				
Gender (female)			0.103 [0.02, 0.2]^a^		
Education (High)				1.0 [0.6, 1.3]^b^	
Years since first HD (3+ years)		0.06 [0.01, 0.1]	0.04 [−0.05, 0.1]	0.8 [0.5, 1.1]^b^	
Previous kidney transplant (yes)	−0.05 [− 0.1, − 0.002]	−0.07 [− 0.1, 0.004]	−0.1 [− 0.2, − 0.03]^a^		
Urine output (more)			−0.1[− 0.2, 0.003]^a^		
Weighted Factor 1				1.0 [0.4, 1.5]^b^	0.8 [0.3, 1.2]^b^
Weighted Factor 2					
Weighted Factor 3					

^a^ indicates significance at the 0.05 level, ^b^ indicates significance at the 0.01 level

Better knowledge of the causes of common intradialytic signs and symptoms was linked to younger age, higher education, receiving dialysis for a greater number of years and rating staff understanding of fluid management higher. Younger patients and patients who rated staff understanding of their fluid management higher were predicted to report more common intradialytic signs and symptoms if they were to experience them.

### Exploring differences between renal units

There were significant differences between Renal Units on all outcomes in unadjusted analysis (ANOVA), which remained when significant predictors were controlled for (Fig. 1).

## Discussion

By surveying patients’ views on target weight management and ultrafiltration in haemodialysis we have focused on a largely neglected but nevertheless important topic. Patients were generally satisfied with their care and felt they had significant influence over decisions, yet the data clearly shows that a large proportion of patients lack knowledge and have misunderstandings about the fundamentals of target weight management and prescription of ultrafiltration volumes. This highlights an opportunity to improve involvement in decision-making, which may improve achievement of target weights and associated outcomes [17].

One in five patients could not recall their target weight, and similar numbers did not know how ultrafiltration volumes were calculated. Important gaps in knowledge about the causes of common signs and symptoms were evident across the population, with younger age, increased education and increased time on dialysis being associated with better knowledge. Almost half of surveyed patients limit ultrafiltration volumes based on previous unpleasant experiences. It is also notable that 64 and 54% of patients felt they were aware of the long term impact of regularly not removing enough fluid or removing too much respectively. Patients also overwhelmingly felt that they are consulted and even given the final say in how much fluid is removed at each session. This strongly highlights the need to address knowledge gaps and contextualize previous experiences to ensure that patients make a truly informed decision when influencing ultrafiltration targets.

Our results are broadly consistent with previous work. The proportion of patients knowing their target weight is similar to findings in US patients [28] and the differences between units reflect international differences [29]. Younger age and higher education have also previously been linked to patients’ knowledge of haemodialysis [30]. However, unlike our results, a study of Norwegian patients suggested some patients feel their input into decisions around fluid removal on HD is not considered [31].

Relatively few patients felt that they would report signs and symptoms of fluid volume disturbance to staff. Not only will this directly impact on managing the patient’s target weight, but symptom reporting has been shown to prompt partnership between patients and the dialysis team [32, 33]. A formal approach to incorporating patient reported symptoms into fluid management decisions, such as the Recova® tool [34], may help to improve regular and systematic input of patient symptoms into decision making. Our results suggest that underreporting of symptoms is associated with older age and having less confidence in the clinical team. Previous work similarly noted that limited or conflicting staff knowledge of the causes of symptoms could reduce reporting of HD-related symptoms and that this could contribute to patients feeling uncertain about the causes of their symptoms [35].

UK guidelines acknowledge the importance of patients understanding their symptoms and how they can be managed and also recommend that healthcare professionals routinely ask patients about their symptoms and explain possible causes of these [36]. Although we did not collect data on healthcare professionals’ practice, it is clear from the patients’ perspective that they may not consider there is routine dialogue around symptoms or good understanding of their cause.

Three factors were generated by the factor analysis. Patients generally perceived that staff understood their fluid management, although non-Caucasian patients to a lesser extent. Difficulties in developing patient-clinician relationships has been reported by patients from non-Caucasian communities [37], including UK haemodialysis patients [38]. Whilst language barriers have been reported as one possible factor, our study excluded patients with language barriers, highlighting the need for further exploration. Considering the second factor, older patients felt less in control of their fluid management, similar to previous findings [30]. Finally, female gender, previous kidney transplantation and lower urine output were associated with greater knowledge of the long term effects of poor fluid management. Interestingly, these were different variables to those associated with the objective test of knowledge about common dialysis symptoms.

A survey to assess UK clinician practice patterns highlighted significant differences between renal units in fluid management [16] and some of the results for units from this study can be seen in Table 5. It is therefore unsurprising that we demonstrated significant between-unit differences in factor scores, knowledge and reporting of signs and symptoms (Fig. 1). Acknowledging the role that the patient has in this process in local standard operating procedures and national guidelines could work towards reducing these differences.

**Table 5:** Practice patterns of fluid management in the renal units from this survey, using data from the study by Dasgupta et al. (16). Renal units 5, 7 and 10 did not participate in the practice patterns study. BCM is Body Composition Monitor, a bioimpedance device (Fresenius Medical Care), and RBV is relative blood volume monitoring

Survey question	Renal Unit						
	1	2	3	4	6	9	10
Does your unit have an agreed policy /guidelines for fluid management?	Yes	No	No	Yes	No	No	No
Is the fluid status of an HD patient routinely assessed on the dialysis unit?	No	Yes	Yes	Yes	No	Yes	Yes
Who is mainly responsible for the routine assessment of an HD patient’s fluid status?	N/a	Senior nurse	Senior nurse	Senior nurse	N/a	Senior nurse	Nurse
Who is mainly responsible when there are concerns around a patient’s fluid status?	Senior nurse	Nephrologist	Doctor	Senior nurse	Nephrologist	Nephrologist	Nephrologist
Frequency of routine dry weight review in the absence of clinical concerns?	Quarterly	Quarterly	Quarterly	Weekly	Monthly	Monthly	Weekly
Do you use technology to assess fluid status?	BCM	RBV	No	RBV	RBV	No	RBV
How do you have a strategy for advice on fluid and salt restrictions?	No	Yes	Yes	No	Yes	No	Yes

UK Patient Reported Experience Measures have shown that “shared decisions around your care” is one of the lowest rated aspects of care in haemodialysis [39] and we know it is a priority area for kidney patients and nephrologists [40, 41]. Good participation in care for dialysis patients is at its core based on information sharing [42, 43]. Patient expertise can be facilitated by patients challenging clinicians’ decisions [42], although patients can find these interactions adversarial and confrontational [44]. Allocated time and space for meaningful patient-clinician interactions is clearly needed.

Our results strongly support the need for considered education for patients and for staff who are central to meeting patients’ educational needs [32]. Although broader in scope than fluid management, the SHARE-HD program is a good example of formalized, education-enabled participation in routine haemodialysis care [45].

### Strengths and limitations

This is the first study with a primary objective of understanding patient perspectives of target weight management and ultrafiltration in haemodialysis. We surveyed a large cohort from a broad range of UK Renal Units that was reflective of ethnicities in the UK haemodialysis population as a whole. Nevertheless generalizability was limited by our exclusion of patients without good comprehension of the English language. As the questionnaires were predominately with staff in the near vicinity, we cannot discount an impact upon patients’ responses. Nonetheless, patients did have the choice to complete the questionnaire at home and all questionnaires were anonymous. We also acknowledge the risk of selection bias from the characteristics of patients that research nurses approached and those more likely to be willing to participate, though the age, gender and ethnicity of the included participants in each unit is comparable to the unit population as a whole (Supplementary Table 2).

## Conclusion

We have demonstrated that haemodialysis patients in the UK feel that they are routinely included in decisions regarding fluid removal during dialysis. However, there are significant gaps in patient knowledge and understanding of the fundamentals of fluid management and prescription of ultrafiltration volumes which could lead to sub-optimal care. Differences between units were also demonstrated. Formalizing the role patients have in the decisions, ensuring appropriate patient education is in place and ensuring opportunities for genuine patient-clinician interaction could provide opportunities to address the longstanding challenge to improve fluid-related outcomes in haemodialysis.

## Supplementary Information

**Additional file 1.** Study questionnaire.

**Additional file 2: Supplemental Table 1.** Questions included in each factor and how strongly they load on to the factor.

**Additional file 3: Supplemental Table 2.** Characteristics of study population compared to those of the study unit as reported in the 2018 UK Renal Registry Report (https://www.renalreg.org/reports/data_to_end_2017/).

## Data Availability

The datasets used and/or analysed during the current study are available from the corresponding author on reasonable request.

## Abbreviations

HD: Haemodialysis
IDH: Intradialytic hypotension
ANCOVA: Analysis of covariance
