# Supplementary material for: Patient perspectives of target weight management and ultrafiltration in haemodialysis: a multi-center survey
Source: BMC Nephrol. 2021 May 20;22:188. doi: 10.1186/s12882-021-02399-7 (PMC8138996; doi:10.1186/s12882-021-02399-7)
Supplement: Supplementary file 1 — Additional file 1. Study questionnaire. [file 12882_2021_2399_MOESM1_ESM.pdf]

UNIVERSITY OF LEEDS

The Leeds Teaching Hospitals NHS
NHS Trust

Patient perspectives of fluid management during haemodialysis

This is a short questionnaire about your understanding and experiences of removing fluid during haemodialysis. We hope to learn about patients' understanding of this subject and how much involvement they have in decisions about fluid management.

The survey should take no longer than ten minutes to complete. You do not have to take part. If you do decide to complete the survey, all responses will be anonymous. Your decision to participate or not will not affect your medical care. This research has been reviewed by a Research Ethics Committee who gave their approval. If you have any questions please ask the researcher who approached you. Thank you for taking the time to read this information and considering taking part in this study.

---

1) What is your current target weight? ☐ *kg* ☐ *I don't know*

2) I understand how the staff use my target weight to work out how much fluid will be removed

Strongly disagree	*Somewhat disagree*	*I don't know*	*Somewhat agree*	*Strongly agree*
☐	☐	☐	☐	☐

3) My target weight is:

☐ *Exactly what I would weigh if I had normal fluid status (that is, if my kidneys were working normally)*
☐ *The lowest weight I can get to without having symptoms during dialysis*
☐ *A compromise between having symptoms from too much fluid removal and leaving too much fluid in my body*
☐ *I am not sure*

4) If I am eating well and gain body weight, but my target weight stays the same, I will finish dialysis with:

☐ *More fluid on* ☐ *Less fluid on* ☐ *About the same amount of fluid on* ☐ *I don't know*

5) Have you ever had an unpleasant experience during dialysis due to having too much fluid removed?

☐ *Yes* ☐ *No* ☐ *I don't know*

6) If yes, has this experience affected how much fluid you are willing to remove during a particular session?

☐ *Yes* ☐ *No* ☐ *I don't know*

7) How many litres of fluid would you usually consider too much to take off during a normal dialysis session?

☐ *1-1.9* ☐ *2-2.5* ☐ *2.6-3* ☐ *3.1-3.5* ☐ *>3.5*

8) I think that longer dialysis sessions allow more fluid to be removed than shorter sessions

Strongly disagree	Somewhat disagree	I don't know	Somewhat agree	Strongly agree
☐	☐	☐	☐	☐

9) If I had some symptoms of being fluid overloaded I would be happy to take off more fluid than normal

Strongly disagree	Somewhat disagree	I don't know	Somewhat agree	Strongly agree
☐	☐	☐	☐	☐

10) A number of symptoms that can occur during haemodialysis are listed below. For each one, please indicate whether you think the symptom can be caused by removing too much fluid or by having too much fluid in your body. If you are unsure, please tick 'I don't know'.

	**Removing too much fluid**	**Having too much fluid in your body**	**I don't know**
Swollen tissue	☐	☐	☐
Feeling very tired after dialysis	☐	☐	☐
Dizziness	☐	☐	☐
Increased risk of fistula failure	☐	☐	☐
Shortness of breath	☐	☐	☐
Passing less urine	☐	☐	☐
Thirst	☐	☐	☐
Cramping	☐	☐	☐
A rapid drop in blood pressure	☐	☐	☐

11) Please indicate if you would routinely inform the healthcare workers looking after you if you experienced any of these symptoms. Tick all that apply, selecting 'I don't know' if you are unsure.

	**Yes**	**No**	**I don't know**
Swollen tissue	☐	☐	☐
Feeling very tired after dialysis	☐	☐	☐
Dizziness	☐	☐	☐
Shortness of breath	☐	☐	☐
Passing less urine	☐	☐	☐
Thirst	☐	☐	☐
Cramping	☐	☐	☐

12) I am aware of the long term impact to my health of regularly not removing enough fluid

Strongly disagree ☐ *Somewhat disagree* ☐ *I don't know* ☐ *Somewhat agree* ☐ *Strongly agree* ☐

13) I am aware of the long term impact to my health of regularly removing too much fluid

Strongly disagree ☐ *Somewhat disagree* ☐ *I don't know* ☐ *Somewhat agree* ☐ *Strongly agree* ☐

14) I am asked about how much fluid is removed at each dialysis session

Strongly disagree ☐ *Somewhat disagree* ☐ *I don't know* ☐ *Somewhat agree* ☐ *Strongly agree* ☐

15) I am given the final say in deciding how much fluid is removed at each dialysis session

Strongly disagree ☐ *Somewhat disagree* ☐ *I don't know* ☐ *Somewhat agree* ☐ *Strongly agree* ☐

16) I am in control of my fluid management

Strongly disagree ☐ *Somewhat disagree* ☐ *I don't know* ☐ *Somewhat agree* ☐ *Strongly agree* ☐

17) I take an active role in my treatment or engage with a shared care programme

Strongly disagree ☐ *Somewhat disagree* ☐ *I don't know* ☐ *Somewhat agree* ☐ *Strongly agree* ☐ *Not applicable* ☐

18) I would be prepared to remain slightly fluid overloaded if it meant I could finish the dialysis session early

Strongly disagree ☐ *Somewhat disagree* ☐ *I don't know* ☐ *Somewhat agree* ☐ *Strongly agree* ☐

19) Having some flexibility around my fluid and diet restrictions is more important to me than making sure I can get to my target weight every session

Strongly disagree ☐ *Somewhat disagree* ☐ *I don't know* ☐ *Somewhat agree* ☐ *Strongly agree* ☐

20) Trying to limit my salt intake is as important as trying to limit my fluid intake

Strongly disagree ☐ *Somewhat disagree* ☐ *I don't know* ☐ *Somewhat agree* ☐ *Strongly agree* ☐

21) I feel that the staff looking after me **pay enough attention to** my fluid management

Strongly disagree ☐ *Somewhat disagree* ☐ *I don't know* ☐ *Somewhat agree* ☐ *Strongly agree* ☐

22) I feel that the nurses looking after me **understand** my fluid management

Strongly disagree ☐ *Somewhat disagree* ☐ *I don't know* ☐ *Somewhat agree* ☐ *Strongly agree* ☐

23) I feel the doctor in charge of my dialysis **understands** my fluid management

Strongly disagree ☐ *Somewhat disagree* ☐ *I don't know* ☐ *Somewhat agree* ☐ *Strongly agree* ☐ *Not applicable* ☐

24) I feel the dietitian who helps to look after my nutrition and diet **understands** my fluid management

Strongly disagree ☐ *Somewhat disagree* ☐ *I don't know* ☐ *Somewhat agree* ☐ *Strongly agree* ☐ *Not applicable* ☐

25) Information about my fluid management that I receive from my GP or other healthcare professionals is consistent with the information I receive from the renal unit

Strongly disagree ☐ *Somewhat disagree* ☐ *I don't know* ☐ *Somewhat agree* ☐ *Strongly agree* ☐ *Not applicable* ☐

26) Information about my fluid management that I receive from informal sources (such as the internet or other patients) is consistent with the information I receive from the renal unit

Strongly disagree ☐ *Somewhat disagree* ☐ *I don't know* ☐ *Somewhat agree* ☐ *Strongly agree* ☐ *Not applicable* ☐

27) The information I am given at each dialysis session is personally tailored to me and my situation that day

Strongly disagree ☐ *Somewhat disagree* ☐ *I don't know* ☐ *Somewhat agree* ☐ *Strongly agree* ☐

<u>Some questions about you</u>

28) What is your age?

☐ *18-25* ☐ *26-35* ☐ *36-45* ☐ *46-55* ☐ *56-65* ☐ *66-75* ☐ *76+*

29) What is your ethnicity? ☐ *White* ☐ *Black* ☐ *Asian* ☐ *Other*

30) What is your gender? ☐ *Male* ☐ *Female*

31) What is the highest level of education you have completed?

☐ *CSE* ☐ *O Level* ☐ *GCSE grades A-C* ☐ *GCSE grades D-E* ☐ *A Level*
☐ *BTEC* ☐ *HNC* ☐ *Degree / HND* ☐ *Full Apprenticeship* ☐ *City & Guilds*
☐ *NVQ* ☐ *GNVQ Advanced* ☐ *Other (please specify)*

32) Do you have any of these health conditions? ☐ *Heart failure* ☐ *Diabetes*

33) These health conditions have an impact on how much fluid is removed during dialysis

☐ *Yes* ☐ *No* ☐ *Not applicable*

34) How many years is it since you first were treated with haemodialysis?

☐ *Less than 1* ☐ *1-3* ☐ *3-5* ☐ *5-10* ☐ *more than 10*

35) Have you ever had a kidney transplant*?* ☐ *Yes* ☐ *No*

36) How much urine do you pass per day?

☐ *None* ☐ *Less than a cupful* ☐ *More than a cupful*

37) How many sessions of dialysis do you have a week?

☐ *One* ☐ *Two* ☐ *Three* ☐ *More than three*

Is there anything else you feel is important to consider when making decisions about how much fluid to remove during dialysis?

If you have any questions about this research, please contact:
Dr. David Keane, Tel: 0113 206 4119; email: david.keane@nhs.net
