# Supplementary material for: Patient perspectives of target weight management and ultrafiltration in haemodialysis: a multi-center survey
Source: BMC Nephrol. 2021 May 20;22:188. doi: 10.1186/s12882-021-02399-7 (PMC8138996; doi:10.1186/s12882-021-02399-7)
Supplement: Supplementary file 2 — Additional file 2: Supplemental Table 1. Questions included in each factor and how strongly they load on to the factor. [file 12882_2021_2399_MOESM2_ESM.docx]

**Supplemental Table 1**: Questions included in each factor and how strongly they load on to the factor.

| **Factor 1: Patients’ perception of staffs’ understanding of their fluid management** | **Factor 2: Perception of being in Control of own Fluid Management** | **Factor 3: Awareness of possible impact to health due to long-term poor fluid management** |
| --- | --- | --- |
| Question 21: I feel that the staff looking after me pay enough attention to my fluid management (0.811) | Question 14: I am asked about how much fluid is removed at each dialysis session (0.513) | Question 12: I am aware of the long term impact to my health of regularly not removing enough fluid (0.792) |
| Question 22: I feel that the nurses looking after me understand my fluid management (0.834) | Question 15: I am given the final say in deciding how much fluid is removed at each dialysis session (0.918) | Question 13: I am aware of the long term impact to my health of regularly removing too much fluid (0.819) |
| Question 23: I feel the doctor in charge of my dialysis understands my fluid management (0.496) | Question 16: I am in control of my fluid management (0.550) |  |
| Question 24: I feel the dietician who helps to look after my nutrition and diet understands my fluid management (0.536) |  |  |
| Question 27: The information I am given at each dialysis session is personally tailored to me and my situation that day (0.568) |  |  |
