# Supplementary material for: Patient perspectives of target weight management and ultrafiltration in haemodialysis: a multi-center survey
Source: BMC Nephrol. 2021 May 20;22:188. doi: 10.1186/s12882-021-02399-7 (PMC8138996; doi:10.1186/s12882-021-02399-7)
Supplement: Supplementary file 3 — Additional file 3: Supplemental Table 2. Characteristics of study population compared to those of the study unit as reported in the 2018 UK Renal Registry Report (https://www.renalreg.org/reports/data_to_end_2017/). [file 12882_2021_2399_MOESM3_ESM.docx]

**Supplemental Table 2**: Characteristics of study population compared to those of the study unit as reported in the 2018 UK Renal Registry Report (https://www.renalreg.org/reports/data_to_end_2017/)

| **Renal Unit** | **Age (years)** | | **Gender (% male)** | | **Ethnicity (% Caucasian)** | |
| --- | --- | --- | --- | --- | --- | --- |
|  | **Study** | **Unit** | **Study** | **Unit** | **Study** | **Unit** |
| 1 (n=292) | 56-65 | 61.7 | 61 | 61.5 | 73 | 72.8 |
| 2 (n=47) | 66-75 | 68.1 | 57 | 62.5 | 79 | 85.4 |
| 3 (n=182) | 56-65 | 64.5 | 65 | 63.6 | 82 | 72.6 |
| 4 (n=150) | 56-65 | 64.9 | 59 | 57.8 | 77 | 55.9 |
| 5 (n=50) | 66-75 | 69.7 | 56 | 66.7 | 94 | 95.4 |
| 6 (n=84) | 66-75 | 68.8 | 64 | 64.1 | 72 | 67.7 |
| 7 (n=27) | 56-65 | 71.1 | 52 | 71.1 | 81 | 61.1 |
| 8 (n=20) | 66-75 | 69.7 | 80 | 68 | 85 | 89.8 |
| 9 (n=69) | 66-75 | 69.0 | 61 | 61.9 | 88 | 93.6 |
| 10 (n=56) | 66-75 | 67.8 | 59 | 62.5 | 96 | 99.4 |
